# *Sedum middendorffianum* Maxim Induces Apoptosis and Inhibits the Invasion of Human Ovarian Cancer Cells via Oxidative Stress Regulation

**DOI:** 10.3390/antiox12071386

**Published:** 2023-07-05

**Authors:** Ju-Yeon Choi, Miran Jeong, Kijun Lee, Jin-Ok Kim, Wan Hee Lee, InWha Park, Hak Cheol Kwon, Jung-Hye Choi

**Affiliations:** 1 Department of Biomedical and Pharmaceutical Science, Kyung Hee University, Seoul 02447, Republic of Korea; chlwndus001@khu.ac.kr (J.-Y.C.); jeongmr@khu.ac.kr (M.J.); standardlee7@khu.ac.kr (K.L.); nayaajo@khu.ac.kr (J.-O.K.); 2 College of Pharmacy, Kyung Hee University, Seoul 02447, Republic of Korea; 3 Hantaek Botanical Garden, Yongin 17183, Republic of Korea; orni@hantaek.co.kr; 4 Natural Product Informatics Research Center, Korea Institute of Science and Technology (KIST) Gangneung Institute, Gangneung 25451, Republic of Korea; inwha129@kist.re.kr (I.P.); hkwon@kist.re.kr (H.C.K.)

**Keywords:** *Sedum middendorffianum* Maxim, human ovarian cancer, apoptosis, invasion, ROS, MMP

## Abstract

*Sedum middendorffianum* Maxim (SMM) is a Korean endemic plant belonging to the Crassulaceae family. This study aimed to investigate the antitumor effects of the SMM extract on human ovarian cancer cells. Among five endemic plants grown in Korea, the SMM extract showed the most potent cytotoxicity in ovarian cancer cells and had little effect on normal ovarian surface epithelial cells. Furthermore, we revealed that the SMM extract dose-dependently induced apoptosis in human ovarian cancer A2780 and SKOV3 cells. The SMM extract markedly stimulated the activation of caspase-3/8, while the broad-spectrum caspase inhibitor and caspase-8 selective inhibitor significantly reversed SMM extract-induced apoptosis. In addition, the SMM extract significantly inhibited cell invasion and the expression levels of matrix metalloproteinase (MMP)-2 and MMP-9 in ovarian cancer cells. Notably, the SMM extract increased the generation of intracellular ROS, and pretreatment with antioxidant N-acetyl-L-cysteine (NAC) significantly suppressed SMM-induced cytotoxicity and anti-invasive activity. Moreover, NAC treatment reversed the SMM-induced inhibition of MMP-2/9 expression. Taken together, these data suggest that the SMM extract induces caspase-dependent apoptotic cell death and inhibits MMP-dependent invasion via ROS regulation.

## 1. Introduction

Ovarian cancer has the lowest five-year survival rate among all gynecological cancers [1]. Approximately 313,000 women are diagnosed with ovarian cancer annually worldwide. More than 70% of patients with ovarian cancer are diagnosed at an advanced stage when the cancer has significantly metastasized outside the ovary because of the lack of specific clinical symptoms and effective early diagnostic screening [2,3]. Currently, chemotherapy using platinum and taxane is the standard therapy for ovarian cancer. However, most patients relapse and, in most cases, fail to respond to available chemotherapeutic drugs [4]. Therefore, the development of novel therapeutics is urgently required to improve the survival rate of patients with ovarian cancer.

Natural products are a great resource for the development of modern medicines because of their diverse biological activities and low toxicity [5,6]. In particular, natural products derived from native plants in each region are highly valuable for medicinal research [7,8]. Korea has more than 3500 plant species, and approximately 500 species are endemic [9], which presents great potential for discovering their pharmacological activities. In our search for medicinal plants with antitumor activity against ovarian cancer, we focused on five Korean endemic plants, namely *Forsythia saxatilis* Nakai, *F. velutina* Nakai, *Ranunculus crucilobus* H. Lev, *Jeffersonia dubia* Maxim, and *Sedum middendorffianum* Maxim (SMM). There have been no reports on the biological activities of *F. saxatilis* Nakai, *F. velutina* Nakai, and *R. crucilobus* H. Lev. The cytotoxic activity of *J. dubia* Maxim extract against human cervical cancer cells [10] and the antibacterial activity [11] and cytotoxicity of SMM against human leukemia cells [12] have been reported. However, little is known about the antitumor activity of these five plants and their molecular mechanisms of action. In this study, we found that among the five plants, SMM exhibited the most significant cytotoxicity against human ovarian cancer cells but had no effect on normal ovarian cells. Thus, we further investigated the effects of the SMM extract on the apoptosis and invasion of human ovarian cancer cells as well as its underlying molecular mechanism of action.

## 2. Materials and Methods

### 2.1. Sample

The aerial parts of *Forsythia saxatilis* Nakai, *Forsythia velutina* Nakai, *Ranunculus crucilobus* H.Lev, *Jeffersonia dubia* Maxim, and *Sedum middendorffianum* Maxim used in this study were obtained from Hantaek Botanical Garden, Yongin, Gyeonggi-do, Republic of Korea) in June 2017. All plant specimens were authenticated by the botanist Jung Hwa Kang at Hantaek Botanical Garden and deposited as herbarium specimens. The dried materials were extracted with 70% EtOH at room temperature for seven days. The solvent was evaporated in vacuo at 35 °C and stored at −20 °C until use. Stock solution of each extract was prepared by dissolving the dried extracts with 100% dimethyl sulfoxide (DMSO).

### 2.2. Materials

Roswell Park Memorial Institute (RPMI) 1640, fetal bovine serum (FBS), streptomycin, and penicillin were obtained from Life Technologies Inc. (Grand Island, NY, USA). 3-(4,5-Dimethylthiazol-2-yl)-2,5-diphenyl tetrazolium bromide (MTT) was purchased from Thermo Fisher Scientific (cat. # L11939.06, Waltham, MA, USA). N-acetyl-L-cysteine (NAC) was acquired from Sigma-Aldrich (cat. # A9165, St. Louis, MO, USA). 2′,7′-Dichlorodihydrofluorescein diacetate (DCFH-DA, cat. # sc-209391) and inhibitors for caspases-8 (z-IEVD-fmk, cat. # sc-3084) and caspases-9 (z-LEHD-fmk, cat. # sc-3085) were purchased from Santa Cruz Biotechnology (Santa Cruz, CA, USA). Pan-caspase inhibitor (z-VAD-fmk) was obtained from ApexBio Technology LLC (cat. # A1902, Houston, TX, USA). Enhanced chemiluminescence (ECL) reagent was obtained from AbClon Inc. (Seoul, Republic of Korea).

### 2.3. Cell Culture and Cell Viability

Human ovarian cancer cell lines A2780 and SKOV3 were originally obtained from the American Type Culture Collection (Manassas, VA, USA). The immortalized ovarian surface epithelial IOSE80PC cell lines were generated by transfection with SV40-T antigen of ovarian surface epithelial cells provided from N. Auersperg (University of British Columbia, Vancouver, BC, Canada). These cells were cultured in RPMI 1640 supplemented with 5% FBS, penicillin (100 U/mL), and streptomycin sulfate (100 μg/mL) and maintained in a humidified atmosphere of 5% CO_2_ and 95% air at 37 °C. Cell viability was assessed using the MTT assay. Cells were plated in a 96-well plate with 50 μL of RPMI medium at a density of 1.0 × 10^5^ cells/mL in each well. After 24 h of incubation, varying concentrations of extracts dissolved in dimethyl sulfoxide were diluted with culture medium and were added into each well. Following 24 h of incubation, 50 μL of MTT (1 mg/mL stock solution) was added, and the plates were incubated for an additional 4 h. The medium was then removed, and the formazan crystal formed in the cells was dissolved in 50 μL of DMSO per well. The optical density values were measured at 540 nm using a microplate spectrophotometer (SpectraMax; Molecular Devices, Sunnyvale, CA, USA).

### 2.4. Annexin V and PI Double Staining for Apoptosis Analysis

For apoptosis analysis, human ovarian cancer cells were seeded in 60 mm culture dishes with 2 mL of RPMI medium at a density of 1.0 × 10^5^ cells/mL per dish. After 24 h of incubation, the cells were treated with SMM extracts for 24 h. The cells were collected, washed twice with cold PBS, and suspended with 500 μL of binding buffer (10 mM HEPES (4-(2-hydroxyethyl) piperazine-1-ethanesulfonic acid)/NaOH, 140 mM NaCl, 2.5 mM CaCl_2_, pH 7.4). Following staining with 1.25 μL of FITC-conjugated annexin V for 15 min and 10 μL of PI (propidium iodide, 50 mg/mL) for 5 min using the Annexin V-FITC Apoptosis Detection kit (ApoScan kit, Biobud Inc., Gyunggido, Republic of Korea) in a dark place, the mixture was analyzed by Guava^®^ easyCyte flow cytometry (Millipore, Billerica, MA, USA).

### 2.5. Western Blot Analysis

A2780 cells were seeded in 60 mm culture dishes containing 2 mL of RPMI medium at a density of 1.0 × 10^5^ cells/mL in each dish. After 24 h incubation, the cells were treated with SMM extracts for 24 h. The cells were collected and washed twice with cold PBS. Total cellular proteins were extracted using protein lysis buffer (cat. # 17081, iNtRON Bio-technology, Seoul, Republic of Korea) following the manufacturer’s instructions, and protein concentrations were measured by the Bradford assay. The protein extracts were mixed with 5x SDS (sodium dodecyl sulfate) sample buffer and heated for 5 min at 95 °C. The mixture was loaded on a gel for SDS (sodium dodecyl sulfate)-PAGE (polyacrylamide gel electrophoresis). After electrophoretic separation, separated proteins were blotted to PVDF (poly-vinylidene difluoride) membranes. After blocking with 5% skim milk for 1 h, the membranes were incubated overnight at 4 °C with diluted primary antibodies against cleaved-caspase-3 (Cell signaling, cat. # 9661S), cleaved-caspase-8 (BD Biosciences, cat. # 551242), MMP-2 (Cell signaling, cat. # 4022S), MMP-9 (Santa Cruz, cat. # sc-12759), and β-actin (Santa Cruz, cat. # sc-81178) in 1% skim milk (dilution 1:1000). After subsequent washing three times with TBS-T (Tris-buffered saline containing Tween-20), the mem-branes were incubated with anti-mouse-HRP (cat. # 115-035-062) or anti-rabbit-HRP (cat. # 111-035-003) secondary antibody (dilution 1:1000, Jackson ImmunoResearch Laboratories, West Grove, PA, USA) at room temperature for 2 h. Immunoreactive bands were visualized by the ECL kit and detected by Image Quant Las-4000 (Fujifilm Life Science, Tokyo, Japan).

### 2.6. Measurement of Reactive Oxygen Species (ROS)

To measure the levels of reactive oxygen species (ROS), the fluorescent probe 2′,7′-dichlorodihydrofluorescin diacetate (DCFH-DA) was utilized, which is a commonly employed method for quantifying hydrogen peroxide (H_2_O_2_). The oxidation-insensitive DCF was used as a control to ensure that changes in uptake, ester cleavage, and efflux of the probe had not occurred. There was no significant alteration in the fluorescence of cells labeled with the oxidation-insensitive probe. A2780 cells were plated in 60 mm culture dishes with 2 mL of RPMI medium at a density of 1.0 × 10^5^ cells/mL per dish. Following a 24 h incubation period, the cells were harvested through centrifugation after being treated with SMM extracts at specific time intervals. Subsequently, the cells were resuspended in PBS and stained with 20 μM DCFH-DA. The fluorescence intensity was then assessed using Guava^®^ easyCyte flow cytometry (Millipore).

### 2.7. Invasion Assay

For the invasion assays, 1.5 × 10^5^ cells were seeded in 1% RPMI 1640 medium into the upper chamber of an insert coated with Matrigel (cat. # 354234, BD Bioscience, San Jose, CA, USA). The lower chamber was filled with media containing 5% RPMI 1640 medium. Following a 48 h incubation period, the cells that had penetrated through the Matrigel and reached the lower surface of the membrane were fixed with methanol for 10 min and stained with 0.1% (*w/v*) crystal violet (cat. # C0775, Sigma-Aldrich) for 30 min. The cells remaining in the upper chamber were removed using a cotton swab, while the cells on the underside of the filter were counted using an inverted microscope (Olympus, Tokyo, Japan). The count of invading cells was performed by observing five randomly selected fields at ×200 magnification.

### 2.8. High Performance Liquid Chromatography-Mass Spectrometry Analysis

The identification of flavonoids in SMM extract was analyzed by high performance liquid chromatography-mass spectrometry (HPLC-MS). The HPLC-MS analysis was performed on an Agilent 1200 system equipped with 6120 quadrupole MSD (Santa Clara, CA, USA), with the employment of ESI source operated in positive ionization mode. A Phenomenex (Torrance, CA, USA) Luna C18(2) column (150 × 4.6 mm, 5 µm) was utilized to separate the compounds with a mobile phase comprising two solvents, water (A) and methanol (B), both acidified with 0.05% formic acid at a flow rate of 0.7 mL/min. The elution gradient consisted of phase B from 10%–100% in 30 min. The sample was prepared as a solution of 5 mg/mL in methanol and filtered through 0.2 um PVDF syringe filter (Whatman, Kent, UK). The injection volume was 10 uL, and the peaks were detected at 254 nm.

### 2.9. Statistical Analysis

The data are prepared as the mean ± SD. One-way ANOVA or Student’s *t*-tests were performed to identify statistically significant differences. We used GraphPad Prism 8.0.2 software for statistical analyses and displaying the graphs (GraphPad, San Diego, CA, USA). *p*-values less than 0.05 were considered statistically significant.

## 3. Results

### 3.1. SMM Extract Inhibits the Growth of Human Ovarian Cancer Cells

**Figure 1:**
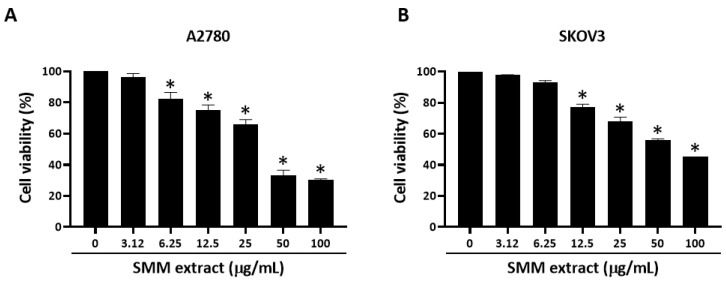
Effect of SMM extract on cell growth in human ovarian cancer cells. Human ovarian cancer A2780 (**A**) and SKOV3 (**B**) cells were treated with SMM extract at the indicated concentrations for 48 h, then an MTT assay was performed to measure the cell viability. Results were highly reproducible in three independent experiments. Data were analyzed using one-way ANOVA followed by Dunnett’s multiple comparison test. * *p* < 0.05 as compared with the untreated group.

First, we performed a cell viability assay to measure the cytotoxic activity of the extracts of five Korean endemic plants, *F. saxatilis* Nakai, *F. velutina* Nakai, *R. crucilobus* H. Lev, *J. dubia* Maxim, and SMM, against human ovarian cancer A2780 cells (Table 1). The extracts of *F. saxatilis* Nakai, *F. velutina* Nakai, and *R. crucilobus* H. Lev showed only mild cytotoxicity, with a median inhibitory concentration (IC_50_) value of less than 100 µg/mL. *J. dubia* Maxim and SMM extracts exhibited significant inhibitory effects on cell growth, with IC_50_ values of 98.35 and 50.25 µg/mL, respectively. Notably, the SMM extract had no cytotoxicity in normal ovarian surface epithelial IOSE80PC cells, while *J. dubia* Maxim extract was more cytotoxic to normal cells (IC_50_ value of 89.20 µg/mL) than to cancer cells. In addition to A2780 cells, the SMM extract exhibited a significant cytotoxic effect on SKOV3 cells in a dose-dependent manner (Figure 1). These results suggest that the SMM extract has significant growth inhibitory activity in human ovarian cancer cells with little cytotoxicity to normal cells. Based on these findings, we chose the SMM extract for further antitumor activity tests.

### 3.2. SMM Extract Induces Caspase-Dependent Apoptotic Cell Death in Human Ovarian Cancer Cells

**Figure 2:**
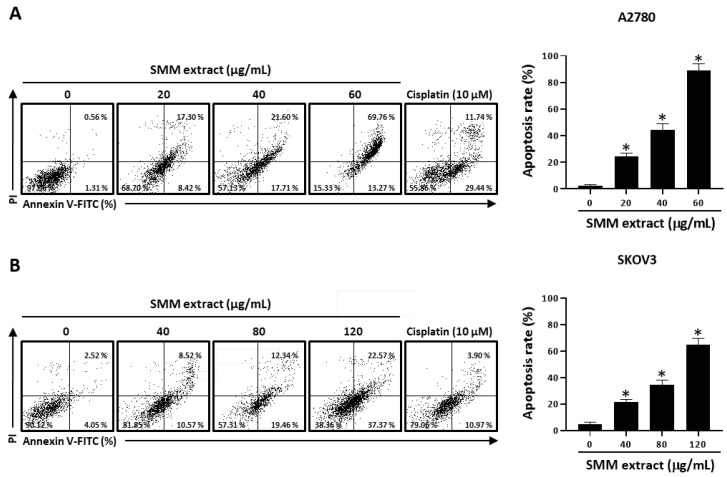
Effect of SMM extract on apoptotic cell death in human ovarian cancer cells. Human ovarian cancer A2780 (**A**) and SKOV3 (**B**) cells were treated with SMM extract for 48 h at the indicated concentrations and stained with PI and annexin V-FITC. The bar graph indicates the percentages of annexin V-positive apoptotic cells in the right quadrants of flow cytometry results. Cisplatin (10 µM) was used as a positive control. The data are representative of three independent experiments. Data were analyzed using one-way ANOVA followed by Dunnett’s multiple comparison test. * *p* < 0.05 as compared with the untreated group.

**Figure 3:**
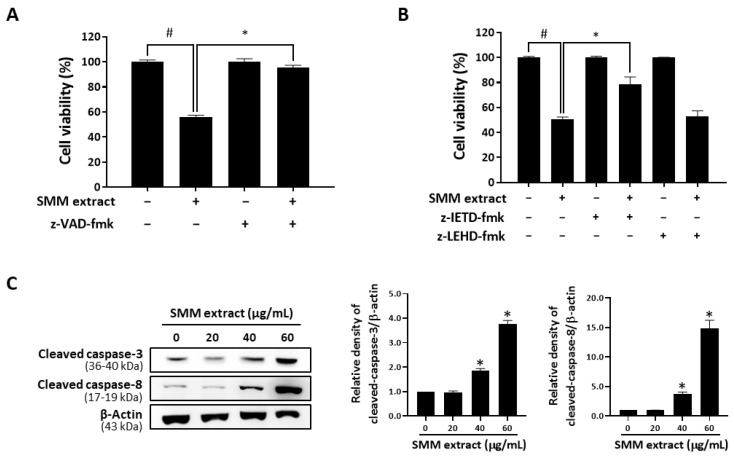
Involvement of caspases in SMM extract-induced cell death in human ovarian cancer cells. (**A**) A2780 cells were pretreated with the broad caspase inhibitor z-VAD-fmk (50 µM) for 2 h, followed by treatment with SMM extract (40 µg/mL) for 48 h. Cell viability was assessed using an MTT assay. (**B**) A2780 cells were pretreated with either the caspase-8 inhibitor z-IETD-fmk (50 µM) or the caspase-9 inhibitor z-LEHD-fmk (75 µM) for 2 h and then exposed to SMM extract (40 µg/mL) for an additional 48 h. Cell viability was measured using the MTT assay. The presented data are representative of three independent experiments. ^#^ *p* < 0.05 indicates significant difference compared to the untreated group. * *p* < 0.05 indicates significant difference compared to the group treated with SMM extract alone. (**C**) A2780 cells were treated with varying concentrations of SMM extract (20, 40, and 60 µg/mL) for 48 h. The levels of cleaved caspase-3 and caspase-8 were analyzed using Western blotting. β-Actin was used as an internal control. The displayed data are representative of three independent experiments. * *p* < 0.05 as compared with the untreated group.

To examine whether the growth inhibitory effect of the SMM extract on human ovarian cancer cells is associated with apoptosis induction, an annexin V-fluorescein isothiocyanate staining assay was conducted with the SMM extract in human ovarian cancer cell lines. As shown in Figure 2A, treatment with the SMM extract (20, 40, and 60 µg/mL) significantly increased the annexin V-positive cell population (25.72, 39.31, and 83.03%, respectively) in A2780 cells. SMM extract also showed dose-dependent apoptosis-inducing activity in SKOV3 cells (Figure 2B). These findings suggest that the SMM extract induces apoptotic cell death in human ovarian cancer cells. Next, we investigated whether the SMM extract-induced apoptotic cell death was associated with the activation of caspases, the well-known key molecules in apoptosis [13,14], in human ovarian cancer cells. SMM extract-induced apoptotic cell death was significantly suppressed in the presence of z-VAD-fmk, a broad caspase inhibitor (Figure 3A). Interestingly, z-IETD-fmk, a selective caspase-8 inhibitor, markedly reversed the inhibitory effect of the SMM extract on cell viability, but z-LEHD-fmk, a selective caspase-9 inhibitor, did not (Figure 3B). In addition, Western blotting analysis revealed that the SMM extract induced the activation of caspases-8, a key player in the extrinsic apoptosis pathway, and an effector caspase, caspase-3, resulting in an increase in the expression levels of their cleaved forms (Figure 3C). These results indicate that the SMM extract can induce caspase-dependent apoptosis in human ovarian cancer cells via the extrinsic pathway.

### 3.3. SMM Extract Increases the Intracellular Levels of Reactive Oxygen Species (ROS), which are Associated with SMM-Induced Apoptotic Cell Death in Ovarian Cancer Cells

**Figure 4:**
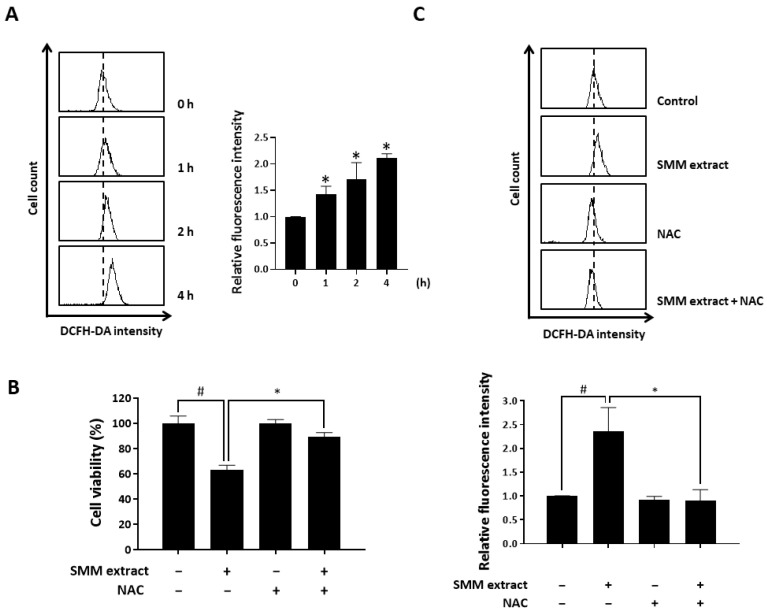
Involvement of ROS in SMM extract-induced cell death in human ovarian cancer cells. (**A**) A2780 cells were treated with SMM extract (40 µg/mL) for the indicated times (1, 2, and 4 h). The cells were stained with DCFH-DA and analyzed by flow cytometry. * *p* < 0.05 as compared with the 0 h treated group. (**B**) A2780 cells were pretreated with NAC (5 mM) for 30 min and then exposed to SMM extract (40 µg/mL) for 48 h. Cell viability was measured using the MTT assay. (**C**) A2780 cells were pretreated with NAC (5 mM) for 30 min and then exposed to SMM extract (40 µg/mL) for 48 h. The cells were stained with DCFH-DA and analyzed by flow cytometry. The data are representative of three independent experiments. ^#^ *p* < 0.05 as compared with the untreated group. * *p* < 0.05 as compared with the group treated with SMM extract alone.

The accumulation of intracellular ROS induces cell death and apoptosis [15]. To assess the impact of the SMM extract on intracellular ROS levels, we utilized the fluorescent oxidation probe DCFH-DA and oxidation-insensitive probe as a negative control. As illustrated in Figure 4A, treatment with the SMM extract resulted in an increase in ROS levels, as indicated by the enhanced fluorescence observed after staining the cells with DCF-DA. In addition, SMM extract-induced cell death was significantly suppressed in the presence of the antioxidant N-acetyl-L-cysteine (NAC) (Figure 4B), and NAC pretreatment significantly reversed the SMM extract-induced ROS accumulation (Figure 4C). These data suggest that the SMM extract induces apoptosis via the regulation of intracellular ROS levels in human ovarian cancer cells.

### 3.4. SMM Extract Inhibits Cell Invasion and MMP Expression in Human Ovarian Cancer Cells

**Figure 5:**
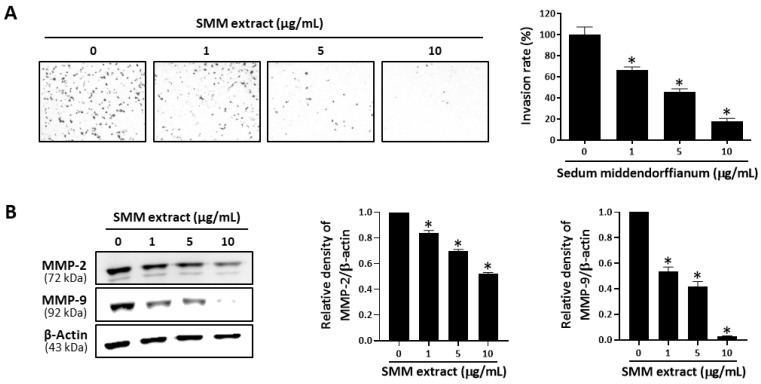
Effect of SMM extract on invasion activity in human ovarian cancer cells. (**A**) A2780 was seeded in Matrigel-coated chambers for the invasion assay and incubated for 48 h in the absence or presence of SMM extract (1, 5, and 10 μg/mL). (**B**) The protein levels of MMP-2 and MMP-9 were determined by Western blot analysis. β-Actin was used as an internal control. The data are representative of three independent experiments. * *p* < 0.05 as compared with the untreated group.

Metastatic properties are the leading cause of human ovarian cancer-related fatalities [16,17]. Therefore, we further investigated the effect of the SMM extract on ovarian cancer cell invasion. As shown in Figure 5A, the SMM extract treatment inhibited the invasion of A2780 cells in a dose-dependent manner. Matrix metalloproteinase (MMP)-2 and MMP-9 play important roles in various steps of metastasis in human ovarian cancer cells [18]. Thus, we investigated the effect of the SMM extract on the expression levels of MMP-2 and MMP-9. Western blotting analysis revealed that treatment with the SMM extract significantly suppressed the expression levels of MMP-2 and MMP-9 in A2780 cells (Figure 5B). These results suggest that the SMM extract inhibits ovarian cancer cell invasion by downregulating MMP expression.

### 3.5. Anti-Invasive Activity of SMM Extract is Associated with Intracellular ROS Levels

**Figure 6:**
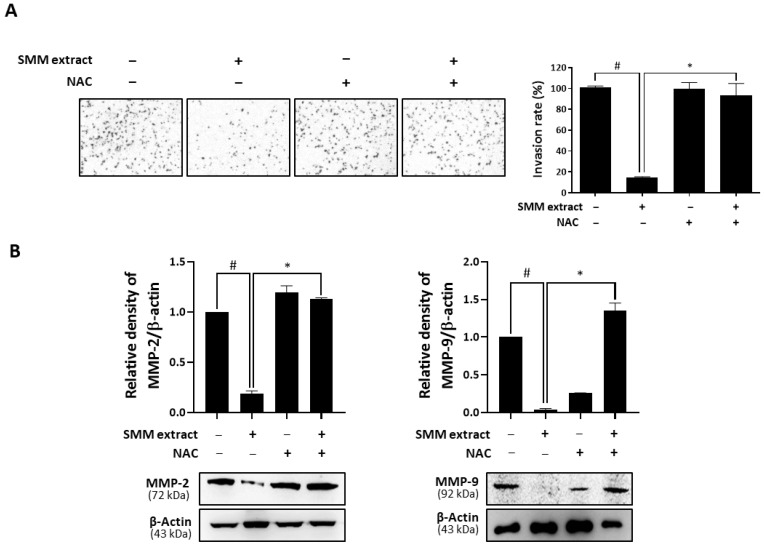
Involvement of intracellular ROS in anti-invasive activity of SMM extract against human ovarian cancer cells. (**A**) A2780 cells were treated with SMM extract (5 μg/mL) and seeded in Matrigel-coated chambers for the invasion assay. The cells were incubated for 48 h in the absence or presence of NAC (5 mM). (**B**) A2780 cells were treated with SMM extract (5 μg/mL) for 48 h in the absence or presence of NAC (5 mM). The protein levels of MMP-2 and MMP-9 were determined by Western blot analysis. The data are representative of three independent experiments. ^#^ *p* < 0.05 as compared with the untreated group and * *p* < 0.05 as compared with the group treated with SMM extract alone.

Intracellular ROS are suggested to have a complex correlation with the metastatic ability of cancer cells [19]. Therefore, we tested whether ROS were associated with the inhibitory effect of the SMM extract on ovarian cancer cell invasion. As shown in Figure 6A, pretreatment with NAC significantly recovered the invasion rate suppressed by SMM extract treatment in ovarian cancer cells. We further investigated the effect of NAC on the downregulation of MMP-2 and MMP-9 expression levels by the SMM extract. The reduced levels of MMP-2/9 by SMM extract were significantly reversed by pretreatment with NAC (Figure 6B). These findings suggest that intracellular ROS accumulation induced by the SMM extract results in the inhibition of MMP-2/9 expression and invasion in human ovarian cancer cells.

### 3.6. HPLC-MS Analysis of SMM Extract

**Figure 7:**
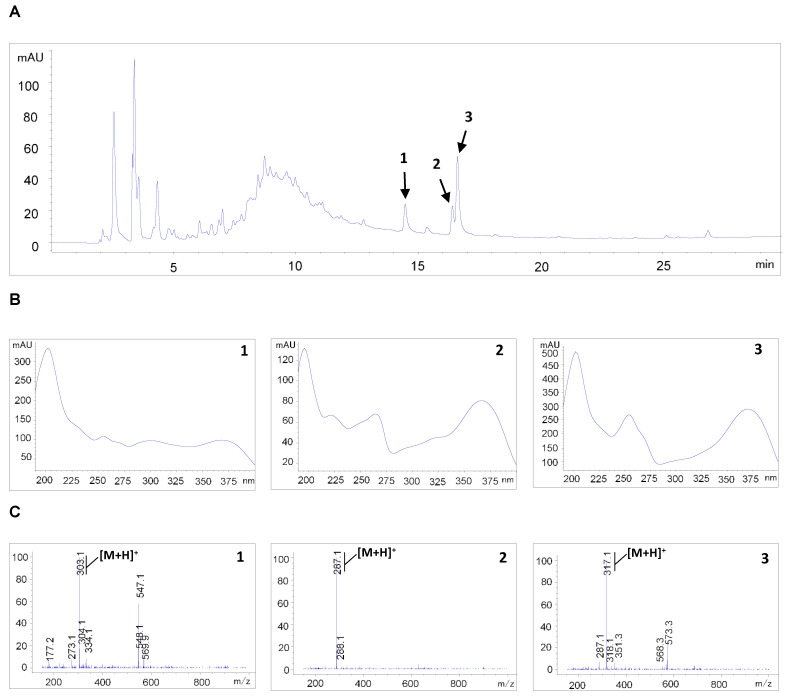
HPLC-MS analysis of SMM extract. (**A**) HPLC chromatogram at 254 nm of SMM extract. (**B**) UV spectra of peaks 1–3. (**C**) MS data of peaks 1–3. Peak assignments: 1. quercetin; 2. kaempferol; 3. isorhamnetin.

Flavonoids in the SMM extract were identified by comparing their elution order, UV spectra, and MS data with those of compounds from the same genus [20,21]. The HPLC-MS chromatogram of the SMM extract displayed peaks (1–3) indicative of the presence of flavonoids, as confirmed by the UV spectrum (Figure 7A,B). Three flavonoids corresponding to protonated molecular ions at *m/z* 303, 287, and 317 were assigned as quercetin (peak 1), kaempferol (peak 2), and isorhamnetin (peak 3), respectively (Figure 7C).

## 4. Discussion

Over the past decades, plants have become a key source for the discovery of novel effective drugs for cancer treatment [22,23]. In particular, endemic plants are attracting attention, as they have a unique phytochemical composition owing to their endemism [24,25,26]. Among the five endemic Korean plants used in this study, we found that the SMM extract had antitumor potential by inducing apoptosis and inhibiting invasion of human ovarian cancer cells. SMM, termed as “aegi-gilincho” in Korea, is an endemic plant species belonging to the *Sedum* genus in the Crassulaceae family [27]. Approximately 121 *Sedum* species (91 endemic) occur in Asia. There are several reports on the antitumor activities of different *Sedum* species. For example, *S. sarmentosum* Bunge and *S. emarginatum* Migo extracts induce apoptosis in HepG2 liver cancer cells [28,29]. *S. oryzifolium* extract and its constituents inhibit the invasion of oral squamous cell carcinoma cells [30]. However, little is known about the antitumor activity of *Sedum* species in Korea, similar to the other SMM members, such as *S. kamtschaticum* Fisch (“gilincho” in Korean) and *S. takesimense* Nakai (“sum-gilincho” in Korean). In a screening study evaluating the cytotoxicity of 280 Korean plants against leukemia cells, SMM methanol extract at 10 μg/mL showed the most potent cytotoxicity [12]. The antibacterial activity of SMM has been recently reported [11]. However, little is known about its antitumor activity and molecular mechanism of action. In this study, we demonstrated for the first time that the SMM extract induces apoptosis and inhibits the invasion of human ovarian cancer cells.

Previous phytochemical investigations of SMM have identified the presence of kaempferol, quercetin, myricetin, and arbutin [31]. In our study, as depicted in Figure 7, the SMM extract used exhibited the presence of the flavonoids quercetin, kaempferol, and isorhamnetin. Notably, quercetin has been suggested to induce intracellular ROS production and display antitumor activities against human ovarian cancer cells [32,33,34]. Considering that elevated ROS levels are necessary for SMM-induced apoptosis and invasion of ovarian cancer cells, it is plausible that the antitumor effects of SMM are mediated by quercetin. It is important to note that plant-derived substances, including the flavonoids, often exhibit low bioavailability due to factors such as instability, poor absorption, and excessive metabolism [35]. Despite their limited bioavailability, many flavonoids have shown activity at low plasma levels [36]. To overcome this challenge, various approaches such as chemical derivatization and innovative delivery systems, including nanoparticles and encapsulation techniques, have been explored to enhance bioavailability. Further investigations are necessary to identify major active components of the SMM extract responsible for its antitumor activity in human ovarian cancer cells. Subsequently, animal studies aimed at improving and optimizing the bioavailability of these compounds should be conducted to explore their potential therapeutic applications.

Apoptosis is the most evolutionarily conserved form of programmed cell death [37]. Caspases play a key role in this process [13]. Caspase-dependent apoptotic cell death is mostly activated by two signaling pathways [38]. Caspase-8 is associated with the death receptor-mediated extrinsic pathway, whereas caspase-9 is an initiator caspase for the mitochondrial-mediated intrinsic pathway. The activation of effector caspase-3 is performed by initiator caspases, such as caspase-9 and caspase-8. Due to their key role in apoptosis, caspases have been considered as effective targets in cancer therapeutics [14,39]. Here, we discovered that the SMM extract induced the activation of caspase-8-dependent apoptosis in human ovarian cancer cells.

Intracellular ROS are implicated in various human disorders, including cancer [40]. In normal physiological conditions, cells tightly regulate ROS levels through homeostatic mechanisms [41]. However, excessive ROS can lead to apoptosis by damaging cellular components, such as nucleic acids and cell membranes [15]. Therefore, the induction of apoptosis through intracellular ROS generation has gained attention as a potential strategy for antitumor interventions [42,43]. In our study, we demonstrated that caspase-8-dependent apoptotic cell death induced by the SMM extract was significantly reversed by pretreatment with the ROS scavenger NAC. These data suggest that the ROS production induced by the SMM extract may stimulate the activation of the extrinsic apoptosis mechanism in human ovarian cancer cells. Interestingly, many polyphenols, including flavonoids, exhibit dual roles as both antioxidants and pro-oxidants [44]. Several flavonoids, recognized as powerful antioxidant scavengers, have been reported to generate ROS, thereby inducing apoptosis and cell death in cancer cell lines. For example, resveratrol increases intracellular ROS levels in human ovarian cancer cells, resulting in caspase-mediated apoptosis [45]. Similarly, quinone compounds and camphor-based pyrimidine derivatives induce caspase-mediated apoptosis through ROS generation in various cancer cells [46]. While ROS-induced apoptosis is commonly associated with the mitochondria-dependent intrinsic pathway, recent studies have also indicated its involvement in the extrinsic pathway [47,48,49]. Nevertheless, the mechanisms by which flavonoids stimulate ROS production are poorly characterized. Our research, along with others, has demonstrated that several plant-derived compounds, including flavonoids, induce apoptosis in cancer cells by generating ROS through NADPH oxidases (NOX) [50,51,52,53]. NOXs, transmembrane proteins that produce ROS through the oxidation of NADPH, have been shown to play a crucial role in cell proliferation and tumorigenesis [54]. Further investigations are required to elucidate whether the SMM extract induces ROS generation via NOX.

MMPs are a family of structurally related zinc-dependent endopeptidases capable of collectively degrading all components of the extracellular matrix that play a key role in the metastasis of various cancers [55,56]. In particular, MMP-2 and MMP-9 play critical roles in the metastasis of human ovarian cancer cells [18]. In this study, we found that the SMM extract reduced the expression levels of MMP-2/9 by inducing ROS accumulation in human ovarian cancer cells, resulting in reduced invasiveness of these cancer cells. To date, findings on the effect of intracellular ROS generation on the metastatic potential of cancer cells are conflicting [19,57]. Some natural products exert anti-invasive activity via ROS-induced downregulation of MMP expression [58,59]. However, the mechanism by which MMP expression is regulated by ROS in cancer cells is poorly characterized. Notably, NOX-mediated intracellular ROS generation is suggested to regulate MMP expression via transcriptional regulation [60,61].

## Figures and Tables

**Table 1:** Cytotoxic activity of five Korean endemic plants in human ovarian cancer cells.

Family	Name	^a^ IC_50_ (μg/mL)	
		A2780	IOSE80PC
Oleaceae	*Forsythia saxatilis* Nakai	165.32 ± 5.98	138.71 ± 5.80
Oleaceae	*Forsythia velutina* Nakai	133.15 ± 10.43	>200
Ranunculaceae	*Ranunculus crucilobus* H.Lév	153.62 ± 4.68	>200
Berberidaceae	*Jeffersonia dubia* Maxim	98.35 ± 6.51	89.20 ± 5.91
Crassulaceae	*Sedum middendorffianum* Maxim	50.25 ± 4.35	>200

Notes: ^a^ IC_50_ is defined as the concentration that results in a 50% decrease in the number of cells compared to that of the control groups. Human ovarian cancer cells were treated with various concentrations of five Korean endemic plants for 48 h, and cell viability was determined using an MTT assay. The values represent the mean ± SD of the results from three independent experiments.

## Data Availability

Data are contained within the article.
